# Genome Sequence of Weissella cibaria M2, a Potential Probiotic Strain Isolated from the Feces of a Giant Panda

**DOI:** 10.1128/MRA.01121-18

**Published:** 2018-09-20

**Authors:** Xin Du, Fuying Dai, Fang Yao, Mingzheng Tan, Qu Pan

**Affiliations:** a Department of Pathogenic Biology, School of Basic Medical Science, Chengdu Medical College, Chengdu, Sichuan, China; Indiana University Bloomington

## Abstract

Here, we report the complete genome sequence of Weissella cibaria M2, a potential probiotic strain isolated from the feces of a giant panda (Ailuropoda melanoleuca). The genome consists of one chromosome of 2.56 Mb and two plasmids. The genome contains 2,420 genes which make up 86.17% of genome.

## Abstract (precis)

Here, we report the complete genome sequence of Weissella cibaria M2, a potential probiotic strain isolated from the feces of a giant panda (Ailuropoda melanoleuca). The genome consists of one chromosome of 2.56 Mb and two plasmids.

## ANNOUNCEMENT

Weissella cibaria is a Gram-positive rod-shaped nonmotile bacterium belonging to the lactic acid bacteria. It is an important microorganism involved in food fermentation and is widely used in fermented food (1). Furthermore, several strains of W. cibaria have been widely researched for their probiotic potential. Besides their general probiotic properties (adhesion and bile salt resistance) (2), these microorganisms can also confer protection against bacterial and fungal infection through bacteriocinogeny, inhibition of colonization, and prevention of inflammation (3–6). Additionally, they are known to be effective in maintaining host health by their antitoxicity, antitumor activity, and immunomodulatory properties (7–11).

Genome sequencing of W. cibaria M2 was performed to identify specific genetic features of this strain and to elucidate its probiotic potential. W. cibaria M2 was grown in MRS broth at 37°C for 24 h prior to genomic DNA extraction using a minibest bacterial genomic DNA extraction kit (TaKaRa, Dalian, China). The extracted genomic DNA was quality checked with the 2100 bioanalyzer (Agilent Technologies, Palo Alto, CA) and sheared into smaller fragments of a desired size with Covaris S/E210 or Bioruptor. Then, a 270-bp insert library with a read length of 2 × 150 bp and a 10-kb template library were constructed for an Illumina HiSeq 4000 instrument and the PacBio RS II platform, respectively. Finally, the libraries were used for sequencing at the Huada Gene Research Center (Beijing, China). Single-molecule real-time (SMRT) sequencing generated 809,092,767 bp of clean data, with a total of 83,136 subreads and a mean read length of 9,732 bp after filtering. The subreads were used for *de novo* assembly with the Hierarchical Genome Assembly Process (HGAP) version 3 in SMRT Analysis software version 2.3.0 (12), which yielded three contigs with an *N*_50_ value of 2,556,971 bp. Correction of the PacBio assembly was performed with soapSNP and soapIndel software using 455 Mb of clean data, which were obtained from the Illumina HiSeq 4000 sequencing after filtering. The single-base quality of the genome reached 0.9999. Gene prediction was managed with Glimmer version 3.02. The functional annotation was accomplished with BLAST with the nonredundant (NR), Swiss-Prot, TrEMBL, Clusters of Orthologous Groups (COG), KEGG, and gene ontology (GO) databases.

The complete genome of W. cibaria M2 consists of a circular chromosome of 2,556,971 bp and two circular plasmids (31,447 bp and 22,296 bp), with G+C contents of 44.89%, 43.60%, and 39.60%, respectively. The genome contains 2,420 genes; the total length of the genes is 2,249,736 bp, which makes up 86.17% of the genome. The number of tandem repeat sequences is 71; the total length of tandem repeat sequences is 21,958 bp, which makes up 0.84% of the genome. Also, 29 minisatellite DNAs, 2 microsatellite DNAs, 91 tRNAs, and 28 rRNAs were predicted. The genome information presented here will help further specific studies of this strain and to exploit its probiotic potential.

### Data availability.

This genome sequence was deposited in GenBank (BioProject number PRJNA381106; GenBank accession numbers CP020928, CP020929, and CP020930; and SRA accession number SRP156997). The version described in this paper is the first version.
